# Fluorescence Detection of Pyrene-Stained *Bacillus amyloliquefaciens* MHR24 in Tomato (*Solanum lycopersicum* L.) Stem Tissues

**DOI:** 10.3390/ijms27094013

**Published:** 2026-04-30

**Authors:** Mónica Hernández-Rodríguez, Gleb Turlakov, Celín Lozano, Eduardo Arias, Alberto Flores-Olivas, Ivana Moggio, José Humberto Valenzuela-Soto

**Affiliations:** 1 Centro de Investigación en Química Aplicada, Departamento de Materiales Avanzados, Boulevard Enrique Reyna Hermosillo 140, Saltillo 25294, Mexico; monica.hernandez.ps@ciqa.edu.mx (M.H.-R.); gleb.turlakov@ciqa.edu.mx (G.T.); celin.lozano.ps@ciqa.edu.mx (C.L.); eduardo.arias@ciqa.edu.mx (E.A.); 2 Departamento de Parasitología, Calz. Antonio Narro, Buenavista, Universidad Autónoma Agraria Antonio Narro, Saltillo 25315, Mexico; aflooli50@gmail.com; 3 SECIHTI-Centro de Investigación en Química Aplicada, Departamento de Biociencias y Agrotecnología, Boulevard Enrique Reyna Hermosillo 140, Saltillo 25294, Mexico

**Keywords:** pyrene markers, rhizobacteria, membrane proteins, pH

## Abstract

The PGPR strain of *Bacillus amyloliquefaciens* MHR24 (MHR24) was recently reported as a strong biocontrol strain. In this study, MHR24 was used to investigate phyllosphere effects during inoculations of tomato leaves (*Solanum lycopersicum* L.). When MHR24 was inoculated on foliar tissue, it caused apical chlorosis symptoms at 3–6 days after infiltration or submersion, which suggests that the bacterium may adopt a potentially pathogenic lifestyle in the phyllosphere. In order to detect the MHR24 interaction with the plant, it was stained with the commercial fluorophore 8-hydroxypyrene-1,3,6-trisulfonic acid trisodium salt, selected from a pyrene series bearing diverse functional groups, based on several in vitro staining assays. Fluorescence used as a detection signal was observed by LSCM mainly in the vascular bundles, suggesting that rhizobacteria may preferentially colonize these tissue regions. Molecular docking, performed by analyzing the possible interactions between the outer membrane protein assembly factor BamB of the family protein *B. amyloliquefaciens* and the fluorophore, indicates that hydrogen bonds with serine 126 (SER126), serine 182 (SER182), isoleucine 180 (ILE180), and tryptophan 66 (TRP66), charges attraction and π-stacking with TRP66, and non-bonded attractions with leucine 224 (LEU224) can occur, which likely gives rise to a stable complex. These results are important in view of the application of MHR24 as part of a sustainable approach for increasing tomato crop production.

## 1. Introduction

It is well documented that plant growth-promoting rhizobacteria (PGPR) offer multiple benefits to crop plants, including the production of phytohormones, biocontrol of pathogens, nutrient solubilization, nitrogen fixation, tolerance to abiotic stress, and the induction of systemic resistance against phytopathogens and pests [1]. Biological control represents an eco-friendly alternative to sustainable agriculture, offering multiple benefits such as low-cost agrochemicals, environmental safety, and enhanced plant health through long-lasting protection against phytopathogens [2,3]. Among the PGPRs, *Bacillus* genus is broadly reported for its growth promotion and biological control abilities [4]. In this sense, the induced systemic resistance is also a mechanism activated by *Bacillus* spp. in host plants, and it is induced in leaves against a broad range of phytopathogens and phytophagous insects [5].

Interestingly, a limited group of beneficial rhizobacteria has been reported to exhibit growth-promoting and biocontrol activities in the plant phyllosphere. In fact, the phyllosphere is a habitat for a diverse array of microorganisms that can exhibit either beneficial or pathogenic lifestyles. However, studies on the phyllosphere with PGPR are scarce [6,7,8].

**Chart 1:**
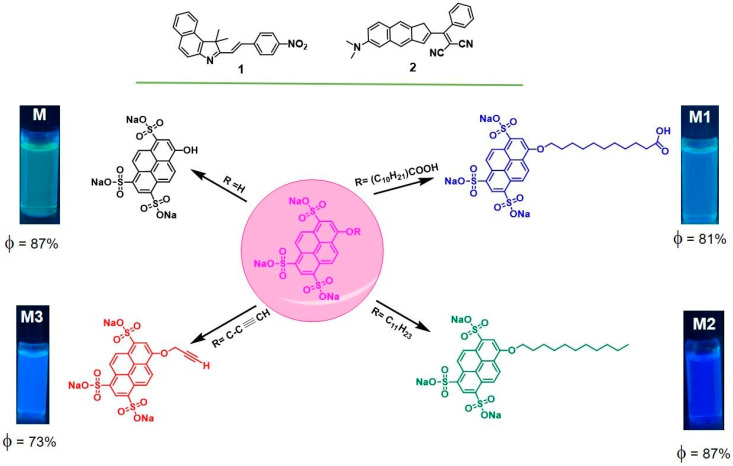
Compounds labelled as **1** and **2** are fluorophores reported in the literature for the detection of rhizobacteria. **M** to **M3** are the fluorophores evaluated in this work. A photo of a dilute water solution under 365 nm irradiation of **M–M3** with the corresponding fluorescence quantum yield ϕ is shown next to each chemical structure.

Under this context, a rapid and highly sensitive detection of rhizobacteria colonization could help their implementation as biofertilizers or biocontrol agents. Typically, root colonization is investigated using GFP (Green Fluorescent Protein) gene integration in PGPR; for instance, *Bacillus amyloliquefaciens* FZB42 *gfp*+ was detected in small zones of the *Arabidopsis thaliana* rhizoplane [9]. However, some limitations were presented by a single copy of the *gfp*+ expression because of the multilayer peptidoglycan composition of the Gram-positive bacteria wall. Additionally, this procedure is quite time-consuming and costly. On the other hand, small molecular fluorophores have been widely proposed as an alternative for staining bacteria of medical interest [10], but few reports have emerged on this strategy for agrobacteria. Chart 1 shows some examples from the literature. Fu et al. [11] synthesized compound **1**, an indole derivative where the cationic charge in the nitrogen is intended to fix the dye to the negative surface of bacteria, while the nitro group is expected to be activated by the redox reaction of the nitroreductase (NTR) flavoprotein that is expressed in many bacteria. The reduction in the nitro group to amine gives a fluorescent molecule that can be used as a sensor for plant diseases, which is demonstrated in *Brassica napus* L. infected with *Xanthomonas campestris* pv. *campestris* (Xcc). Conversely, Jung et al. [12] studied a vast catalog of fluorescent dyes, including derivatives of coumarin, BODIPY, rhodamine, tetraphenylethylene, naphthalene, and fused aromatics for the detection of *Erwinia amylovora*, a pathogen bacterium that causes fire blight infection in several plants, including pears and apples. After a first screening through laser confocal microscopy, they selected naphtobenzofuran **2**, which is a fluorophore with an electron donor and acceptor substituents that gives a strong fluorescence in the presence of the bacteria. The turn-on in the fluorescence was ascribed to the interaction of the dye with lipopolysaccharides and lipoproteins of the outer membrane of the bacteria. Recently, we proposed the pyrene fluorescent markers **M, M1, M2** and **M3** as another approach to bacterial staining and root colonization analysis [13]. The structure of the pyrene dye bears three sodium sulfonate groups at the 1, 3 and 6 positions, and the chemical modulation is given by substituting diverse alkyl chains in the -OH group (8-position) through the Williamson reaction with R = undecanoic acid (**M1**), undecane (**M2**), or a propargyl (**M3**) chain.

In particular, we found that **M1** presented the strongest fluorescence response of the series when in contact with *Bacillus subtilis* LPM1, where a fluorescent biofilm could be detected by Laser Scanning Confocal Microscopy (LSCM). On the contrary, **M2** was the marker to give the best response with *Bacillus subtilis* PY79, forming fluorescent bacterial agglomerates, while **M3** did not interact with any *Bacillus*. None of the tested markers were able to stain the Gram-negative *Escherichia coli* as a model. These results suggest that both **M1** and **M2** markers can be used for the staining and detection of Gram-positive bacteria by fluorescence microscopy. Indeed, the root colonization studies of *B. subtilis* could easily be visualized by LSCM when it was stained with **M1**. It is worth noting that **M** (sometimes called HPTS or pyranine) was used just as a comparative molecule for the intrinsic photophysical properties (in water) but was not evaluated with any bacteria [13] because its fluorescence properties vary with the pH [14], which is variable in the soil where the tomato roots should be analyzed for *B. subtilis* detection. However, for phyllosphere studies, which is the above-ground surface of plants, this limitation does not apply, and **M** can be used.

We recently isolated a PGPR from tomato soil crops grown in open fields, identified as *Bacillus amyloliquefaciens* strain, and labeled as MHR24. MHR24 was found to have biocontrol activity against fungal phytopathogens such as *Alternaria alternata* (Aa), *Botrytis cinerea* (Bc), *Fusarium oxysporum* F1 (F1), *F. oxysporum* F2 (F2), *F. oxysporum* R3 (F3), and *Sclerotinia sclerotiorum* (Sc), which commonly affect tomato crops [15]. Additionally, it promotes plant growth as studied in the model plant *Arabidopsis thaliana* [16]. In the frame of our research on the rapid and easy screening of PGPR in plants in order to follow their colonization patterns and optimize their use as biocontrol agents or biofertilizers for sustainable agriculture, the present work is addressed to investigate whether the appealing properties of MHR24 persist when it is inoculated into the phyllosphere. For this purpose, and following the previous results and literature reports, we used fluorescence as an alternative quick and easy method of detection using the pyrene marker **M**, selected after screening of the fluorescent response with the four pyrene markers. Interestingly, fluorescence was mainly observed in the vascular bundles of tomato plants by Laser Scanning Confocal Microscopy (LSCM), indicating that, presumably, MHR24 could adopt a pathogenic lifestyle in the phyllosphere.

## 2. Results and Discussion

### 2.1. Selection of the Marker Through Fluorescence Assays

**Figure 1:**
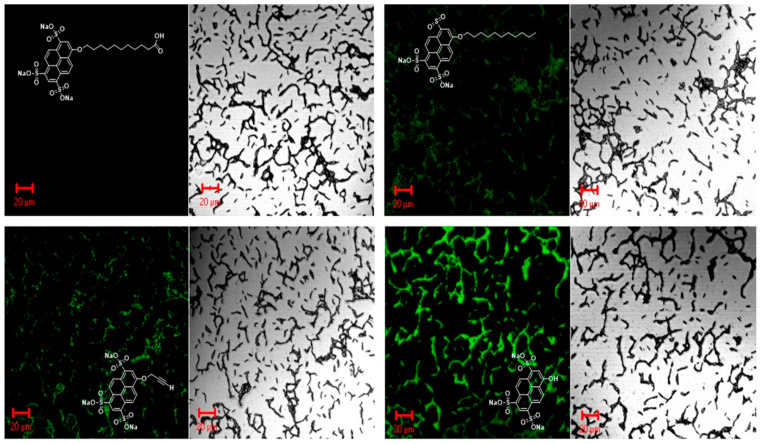
LSCM images of *Bacillus amyloliquefaciens* MHR24 stained with the four pyrene markers (see inserted chemical structure). Fluorescence (**left**) and reflection (**right**). Excitation of Ar line at 488 nm.

As mentioned in the Introduction, in the phyllosphere, no changes in pH occur, so the four pyrene derivatives **M–M3** can be investigated as possible markers for MHR24 detection in the phyllosphere. Thus, the first research task was to select the marker that presents the best fluorescence response with MHR24, for which fluorescent microscopy and spectroscopy studies were conducted for MHR24 after treatment with each of the pyrene compounds. Figure 1 shows the LSCM images of the slide samples of MHR24 in the presence of the four markers (in the following labeled as **MHR24 + M–M3**). In the reflection channel, bacterial aggregation is observed in all samples, whether stained or not (ESI). At least at the magnification that the confocal microscope allows, no structural changes in the bacteria are observed after staining. In the fluorescence channel, **MHR24 + M, MHR24 + M2**, and **MHR24 + M3** (to a minor extent) show fluorescent bacterial aggregates; i.e., the markers seem to form a fluorescent coating on all the bacterial surfaces.

Interestingly, **M1**, which was the pyrene marker that better stained *B. subtilis*, does not exhibit any emission with MHR24 under the tested excitation conditions. Quantitative determination of fluorescence intensity through processing of confocal microscopy images has several drawbacks and possible errors [17]. Moreover, the fluorescence response in the LSCM analysis may depend on: (i) the concentration of the marker deposited on the bacteria; (ii) absorption maxima proximity to the laser excitation; (iii) emission maxima in the detection window; (iv) fluorescence quantum yield. Because of these considerations, we carried out an analysis of the optical properties of the slides by UV–Vis and fluorescence spectroscopy to validate the qualitative fluorescent response of the confocal images and to investigate the origin of the presumable best response with **M**.

**Figure 2:**
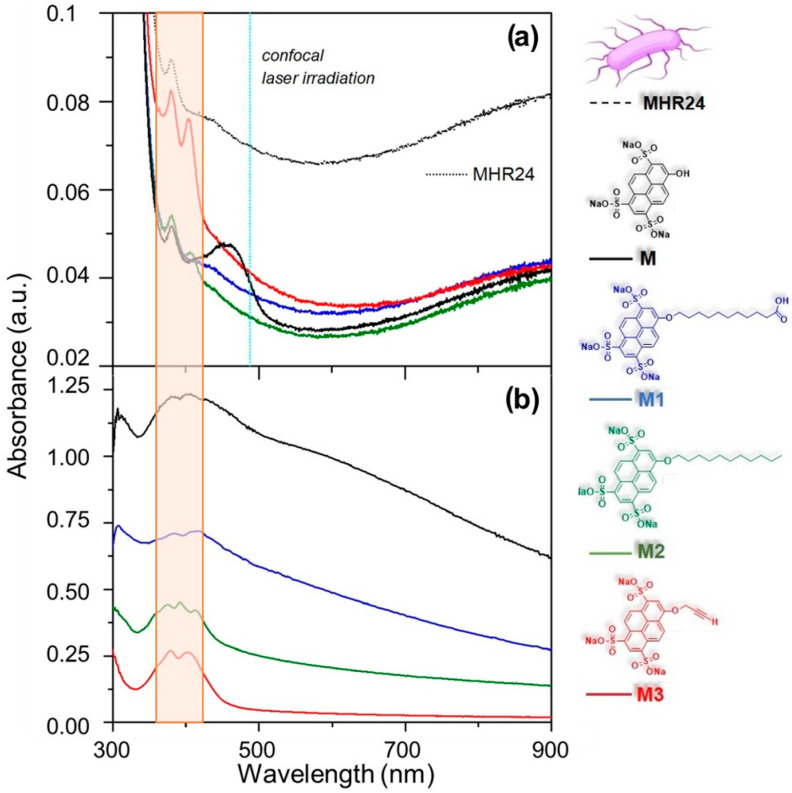
UV–Vis spectra of the confocal microscope samples. (**a**) Pyrene markers **M–M3** in the presence of the MHR24 strain. The spectrum for the unstained MHR24 sample is included for the sake of comparison. Legend indicates the chemical structure of the markers and a sketch of a bacteria for spectra identification. The laser wavelength used for confocal is shown as a cyan line. (**b**) **M–M3** without bacteria.

The UV–Vis spectra of all the samples, as shown in Figure 2 (including the bacteria MHR24 as a reference), present a peak at around 380 nm, which is thus ascribed to the bacteria. The **MHR24 + M1** spectrum is practically identical to that of the bacteria MHR24, corroborating that **M1** barely stains it; the stronger baseline may be due to the light dispersion of the big package of bacteria agglomerates as observed in Figure 1. For **MHR24 + M2** and **MHR24 + M3**, an additional peak appears at 405 nm. This is ascribed to pyrene absorption, as it matches the same bands in the UV–Vis spectra of the marker samples without bacteria (bottom panel and pink rectangle). For **MHR24 + M**, this band shifts to 460 nm, which is in agreement with the absorption peak of **M** in its protonated form [14]. It is important to note that this red shift is closer to the excitation wavelength used in the LSCM analysis (cyan line in Figure 2), which explains the strong fluorescence emission from this sample despite its absorbance being lower than **MHR24 + M3**.

**Figure 3:**
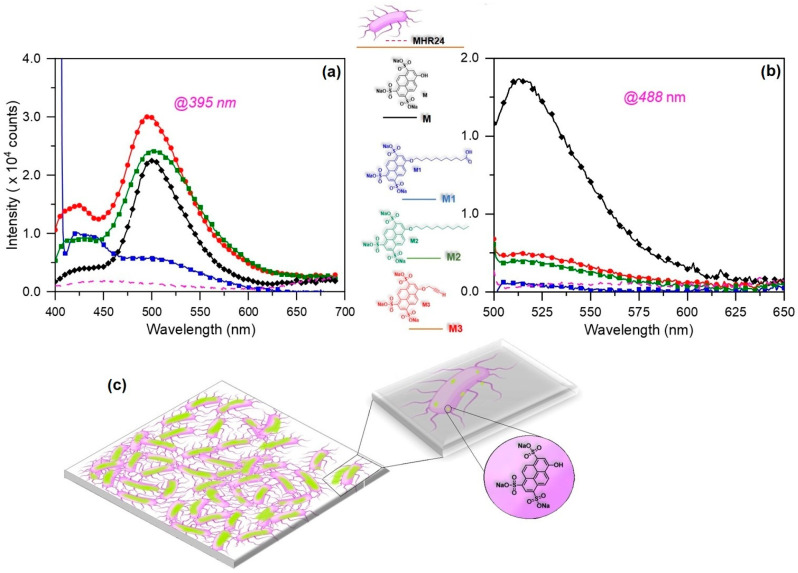
Fluorescence spectra of confocal microscopy samples of *Bacillus amyloliquefaciens* MHR24, unstained or stained with pyrene markers **M** to **M3**, by exciting at 395 nm (**a**) or 488 nm (**b**). Between (**a**) and (**b**), the legend shows the chemical structure of the markers and a sketch of a bacteria for spectral identification. (**c**) Sketch depicting the stained bacteria sample, with **M** as a representative marker.

The fluorescence spectra of the slides were recorded by exciting close to the maxima (at 395 nm) and also at 488 nm, which is the laser excitation available in our confocal microscope. When the excitation is close to the absorption maxima (left panel of Figure 3), all of the spectra present emissions that confirm the presence of the markers. The fluorescence spectra have a maximum peak at around 430 nm, which retains the excitonic features of water solution of the markers [13]. Moreover, another more intense and broader peak is found at ~510 nm. When the excitation is at the wavelength used for laser confocal microscopy (i.e., 488 nm, right panel of Figure 3), just the red-shifted emission is found as the other one is at a higher energy. The intensity is stronger for the sample stained with **M**, confirming the qualitative fluorescence response observed in the confocal images and the fact that this latter sample has an absorption peak close to the excitation wavelength. In order to gain insights about the origin of the emission at 510 nm, we performed further analysis (ESI): (1) The excitation spectra recorded at 510 nm exhibit a band at ~405 nm, as in the absorption spectra; this means that there is only one electronic excited state that is responsible for the absorption and emission. However, for the sample **MHR24 + M**, another strong peak is observed at 464 nm, matching the 460 nm band observed in the UV–Vis spectrum (Figure S1, ESI), which confirms the presence of an additional excited state. (2) The photophysical properties of the precursor suspensions are practically the same for the markers in water solution [13], i.e., excitonic-type, with the main UV–Vis peak at 403 nm and fluorescence at 425–430 nm for **MHR24 + M1** or **MHR24 + M2**, or **MHR24 + M3** and at 513 nm for **MHR24 + M,** corresponding to the emission from the anion species of the pyrene sulfonate (Figure S2, ESI). This means that the marker in the water suspensions with the bacteria are well dispersed as their dilutions and agglomerates arise after solvent evaporation; indeed, the confocal images of control samples also present big agglomerates (Figure 1 and Figure 3) if the pH 7 of the bacteria culture is increased to 10. The fluorescence response as observed in the confocal images (Figure S3, ESI) and the emission spectra of the glass slides (Figure S4, ESI) are similar for all the samples, but for the **MHR24 + M**, it is a bit more intense than at pH 7. For this latter sample, the peak at 430 nm disappears, confirming that at pH7, it is due to the neutral (OH) form. These results indicate that for **M1–M3** markers, the emission peak at 405 nm is monomer-like, while that at 510 nm is likely associated with excimers, as neither the absorption nor the excitation spectrum presents additional peaks due to aggregates. A similar optical response was reported for a fluorescent sensor based on an imidazolium-derived pyrene [18]. In the case of **M**, for which a stronger fluorescence is always observed, and in particular at pH 10, further considerations are to be considered, because, in addition to excimer formation, **M** presents excited state proton transfer (ESPT) that also gives rise to emission at 510 nm [19]. Thus, for this sample, the highest fluorescence at ~510 nm can also derive from ESPT of the pyrene -OH with the bacteria through the anionic components that are found in the cell wall, and that could be more available at pH 10, a feature that is not possible for the other markers, whose emission is weak.

In this respect, it is worth mentioning that the z-potential analysis was performed on MHR24 stained with **M** at neutral pH and compared with MHR24 and **M** as control samples. The marker solution shows a negative Z value (−42.9 ± 0.6 mV), consistent with the three sulfonate groups and the partially dehydrogenated OH (at neutral pH). MHR24 has a greater negative charge (−56.4 ± 1.2 mV) as it is a Gram-positive *Bacillus*, meaning it has a single cell membrane surrounded by a thick layer of peptidoglycan. As a result, the cell surface is negatively charged because of the phosphate groups. The stained sample exhibits practically the same negative value (−57.0 ± 1.0 mV) as the control bacteria, which could indicate the formation of a thin film of **M** on MHR24 or that the marker interactions with the bacterial surface do not change the overall external charge strongly.

### 2.2. Effect of Staining on MHR24 Surface

**Figure 4:**
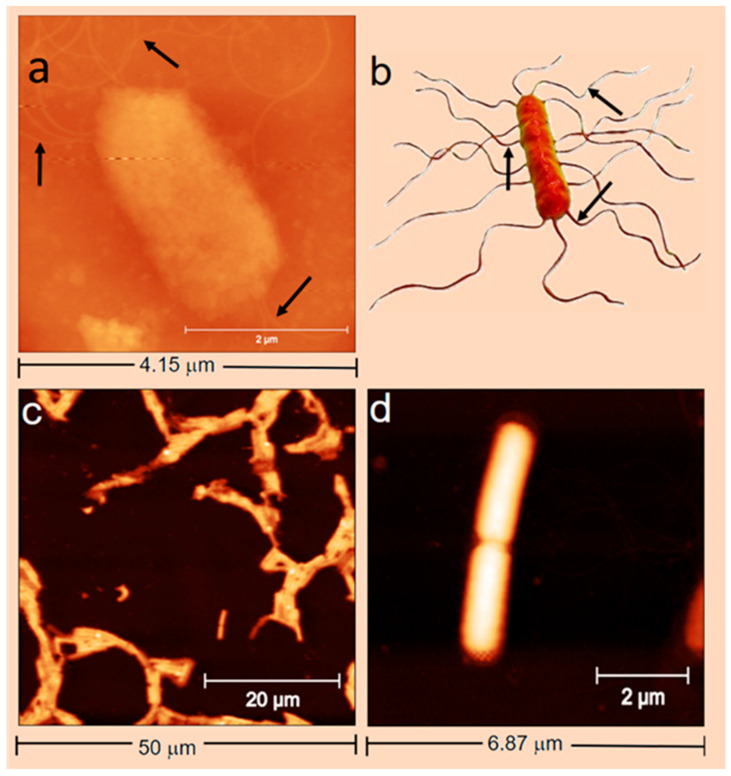
AFM images of MHR24 bacterial cells at pH 7. (**a**) Unstained bacteria where the typical flagella are signaled with arrows, and (**b**) schematic representation of unstained bacteria shown in (**a**). (**c**) Agglomerates of bacteria stained with the pyrene marker **M**; (**d**) scanning in the selected area of image (**c**).

Previous studies have suggested that **M** coats the whole bacterial surface. As this is important for final detection in the phyllosphere, further analyses were carried out on the same slides by atomic force microscopy (AFM) that allows investigation of eventual changes in the bacterial surface at higher magnification than confocal microscopy. Some representative images of the bacteria at pH 7, with or without the marker, are presented in Figure 4 and Figure S5. Figure 4a shows that *B. amyloliquefaciens* MHR24 has the typical *Bacillus* shape, with a uniform surface topography without ruptures or bulges, and average dimensions of 3.20 ± 1.25 µm in length and 1.07 ± 0.29 µm in width. Distinctive peritrichous flagella can be identified (indicated as arrows in Figure 4a and better visualized in the schematic representation of the bacteria, Figure 4b). Similar observations were reported for *B. amyloliquefaciens* FZB42 [20], as well as for other bacteria such as *E. coli*, *Bacillus subtilis*, and *Salmonella enterica serovar Typhimurium*, with a proportion of 5–7 flagella per bacterium [21]. Both the unstained (Figure S5) and the stained bacteria form agglomerates, as can be observed in Figure 4c, confirming the confocal results.

**Figure 5:**
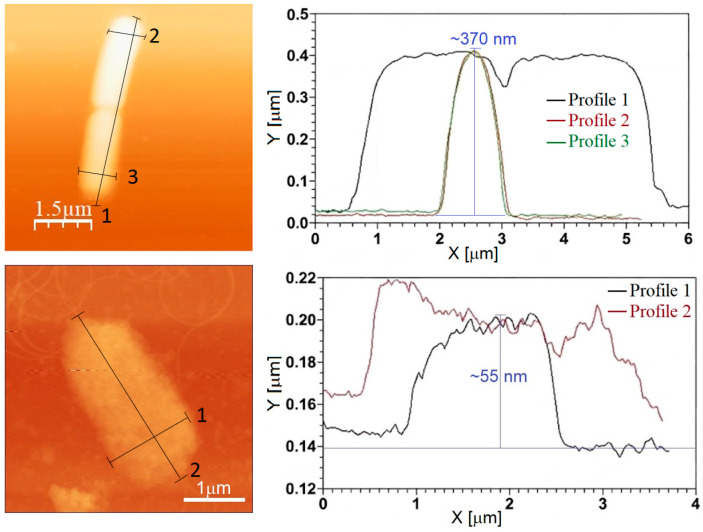
Topographic AFM images of MHR24 (unstained, **bottom**) and **MHR24 + M** (stained, **top**) at pH 7 with corresponding profile graphs determined along the indicated lines. Average heights are indicated.

The corresponding profile graphs (Figure 5 and Figures S6–S8) indicate a significant increase in the average height of the bacteria surface, from 55 nm to 370 nm, after staining with **M**. The presence of the marker can also be confirmed by the increase in roughness, which was estimated by tracing a line along the bacteria (Table S1, ESI). For numerical evidence of the changes in roughness, we used *R_q_* (or RMS), a common topographic parameter corresponding to the height deviation along a mean line, averaged in a scanning area, and calculated as a square root of the mean of squared heights, according to the following equation [22]:

$$R_{q}=\sqrt{\frac{1}{l}}\int_{0}^{l}Z^{2}\left(x\right)dx,$$

where:

*R_q_* = root mean square roughness;
*l* = evaluation length;
*Z*(*x*) = height deviation from the mean line at position *x*.

The *R_q_* is 13.21 nm for untreated bacteria, while for MHR24 with **M**, it is 19.64 nm.

The increase in both parameters, height and *R_q_*, confirms that **M** covers the bacterial surface, forming a rough layer of around 320 nm (thickness with **M**-pristine thickness).

**Figure 6:**
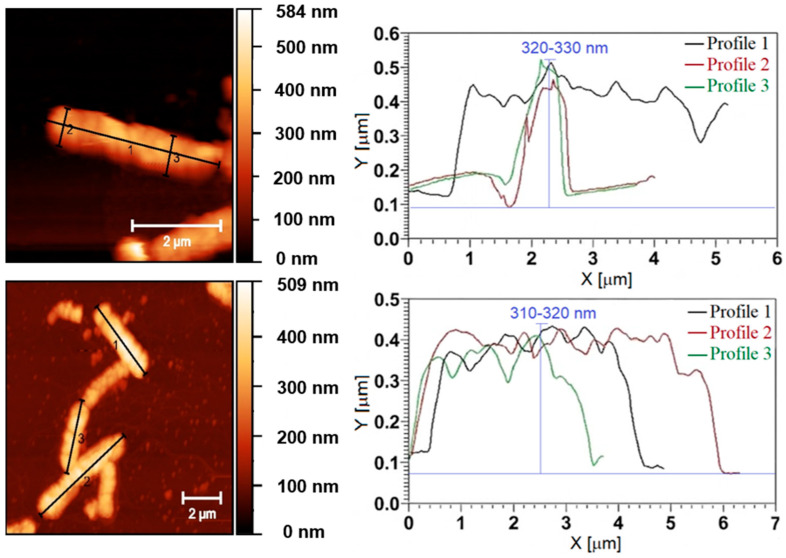
Topographic AFM images of **MHR24 + M** treated at pH 5 (**top**) and pH 10 (**bottom**) with corresponding profile graphs. Average heights are indicated. The color scale represents the surface height, darker tones correspond to lower height regions, whereas lighter tones indicate higher elevations in the sample topography.

To complete the morphological analysis, we also carried out the AFM study on the slides prepared from the stained bacteria culture at different pH. In both the unstained (Figures S5 and S6) and stained (Figure 6 and Figures S5 and S6) bacteria, the AFM images reveal that the cell surface exhibits irregularities and a loss of uniformity with focal depressions and localized indentations in the bacterial envelope, resulting in an irregular topography and loss of structural continuity. These changes are more prominent for higher pH. The heights increase to about 240–250 nm for the unstained bacteria (Figure S7) and to ~320 nm (Figure S8) for the **MHR24 + M** sample. However, *R_q_* has no clear trend. It remained almost constant, with a value of 13.21 nm for MHR24, but decreased to 16.76 nm at pH 5 and increased to 28.98 nm at pH 10 for **MHR24 + M** (Table S1). This seems to suggest that, at the two extreme pH levels, there is a concomitant effect in the roughness of the bacterial surface: the effect of the marker, and of the pH.

**Figure 7:**
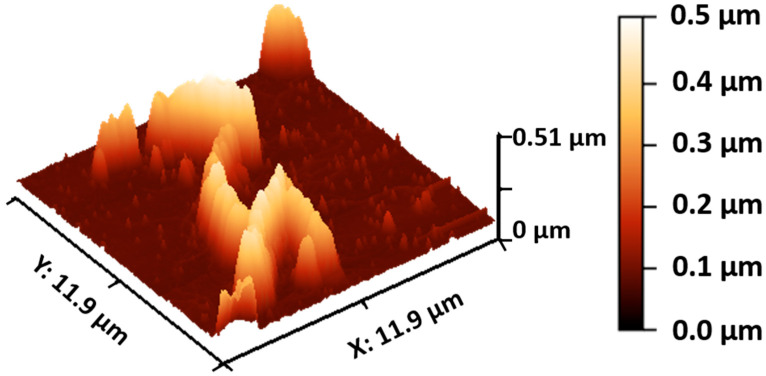
3D AFM image of **MHR24 + M** at pH = 10. The color bar depicts variations in surface height across the sample; darker shades represent lower regions, while lighter shades correspond to areas of greater topographic elevation.

Indeed, at pH 10, damaged cells are clearly presented with wrinkled outer layers. Bacterial interactions with the environment are determined by the physicochemical properties of the cell surface, particularly by the surface charge associated with acid–base functional groups in the bacterial envelope, so at this pH, the bacteria can lose structural integrity and become more sensitive [23,24]. Figure 7 shows a 3D profile of a stained bacterium, where the cell wall has irregular edges. The alterations in growth can be seen as filamentous with a length of 3.83 ± 0.48 µm. It is reported that this effect may be a response to physical, biological, and/or chemical stress in different bacterial genera as a survival strategy [25].

From the AFM, the formation of a continuous film of **M** on MHR24 at pH 7 can be observed, which is the physiological value in the leaves.

### 2.3. Simulation of the Bacteria–Marker Interactions Through Molecular Docking

**Figure 8:**
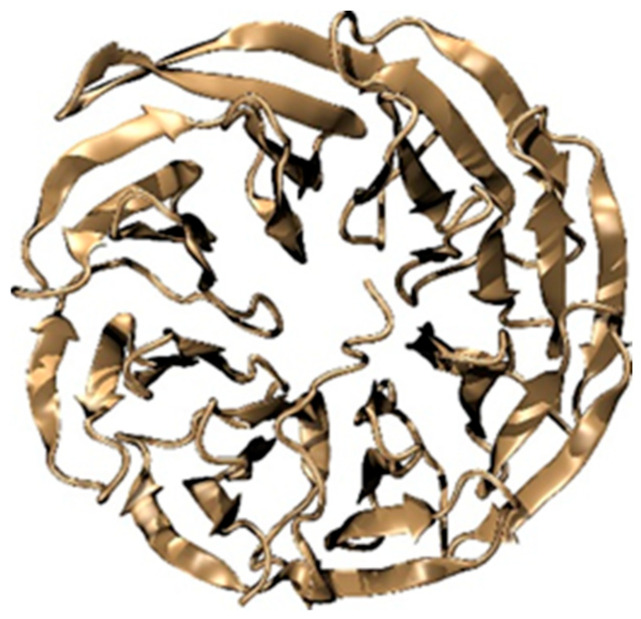
Simulated structure of membrane protein assembly factor BamB family protein from *B. amyloliquefaciens*.

In order to gain insight into the interactions of **M** with the bacteria, which could explain its coating on MH24, molecular docking studies were carried out and revealed that the fluorophore interacts with specific sites on the generated *B. amyloliquefaciens* factor BamB protein. The protein was selected because it has been previously reported as a membrane-associated protein in species, isolated from soil, and with activity PQQ-dependent dehydrogenases [26]. Its subcellular localization and potential involvement in interactions make it a relevant candidate for assessing possible affinities with the target molecule. The BamB family protein adopts a stable propeller-like conformation (Figure 8), showing a binding affinity of 8.83 kcal/mol. It is worth noting that because no experimental structure has been reported for this protein, the whole analysis was based on a computationally modeled structure and its predicted binding sites.

**Figure 9:**
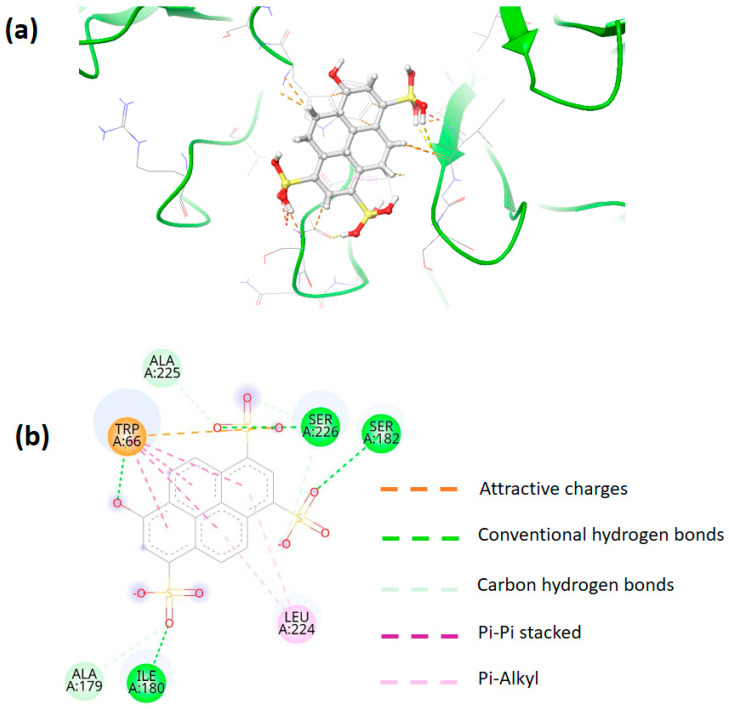
Representation of the interaction between the ligand and the amino acid residues within the protein’s active site. In (**a**), the tridimensional sketch shows the functional groups of M that interact with the protein (green ribbons). In the bidimensional image (**b**), the specific interacting aminoacids of the protein are indicated in the circles and the interactions that stabilize the protein-ligand complex are shown as colored lines, according to the color identification.

Ligand-receptor interaction analysis (Figure 9) shows that the docked fluorophore can be stabilized by a network of different interactions: hydrogen bonds, electrostatic interactions, and π-stacking interactions. The anionic sulfonate group can, in fact, interact with SER126, SER182, ILE180, through hydrogen bonds and with TRP66 through hydrogen bonds and charges attraction. Concurrently, the pyrene ring exhibits π-stacking interactions with TRP66 and non-bonded interactions with LEU224. In general, the presence of multiple stabilizing interactions suggests that the pyrene-based marker may form a stable complex with the protein surface.

### 2.4. Inoculation of MHR24 in Tomato Leaves

**Figure 10:**
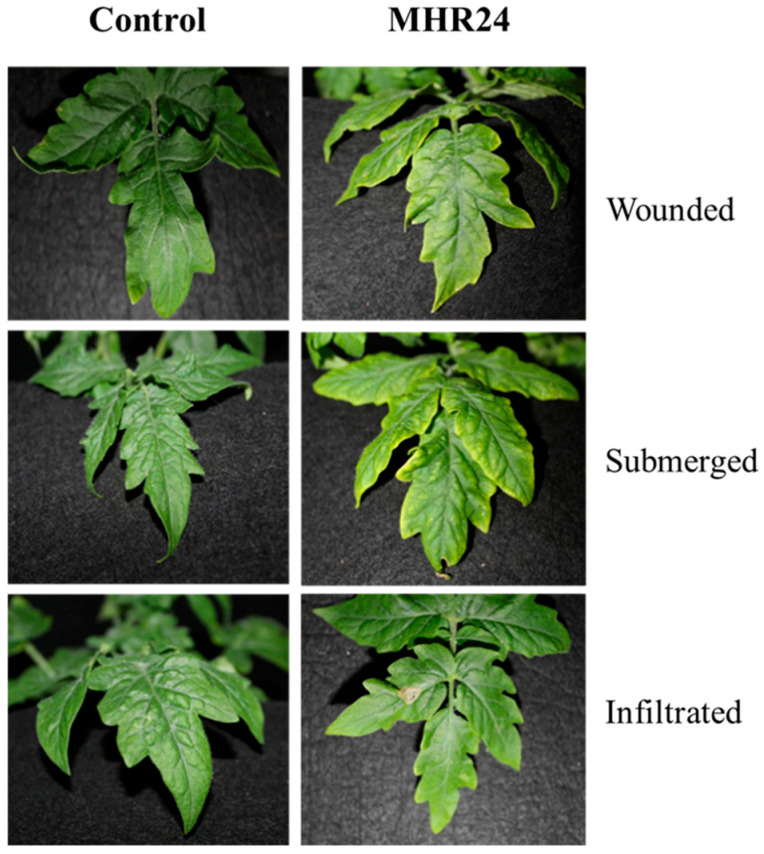
Inoculation of *Bacillus amyloliquefaciens* MHR24 on tomato phyllosphere under growth chamber conditions. Treatments: control, uninoculated, and MHR24, inoculated cotyledon (wounded, submerged, and infiltrated).

Regarding MHR24-phyllosphere interactions, tomato leaves exhibited apical chlorosis after 1 week post-inoculation (folioles submerged, infiltrated, or mechanically wounded) compared with uninoculated control plants (Figure 10). The apical chlorosis observed in tomato leaves was consistently reproduced in independent experiments under growth chamber conditions.

**Figure 11:**
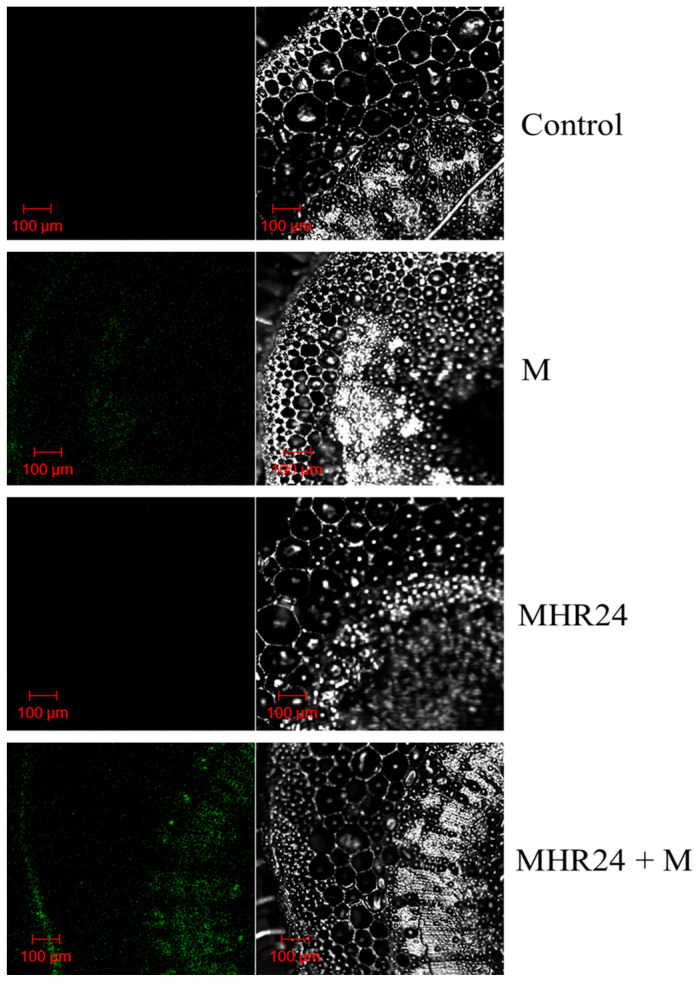
LSCM images of stem tissue collected from tomato plants. **Plant control**, no-stained tissue; **plant + M**, stained with pyrene marker; **plant + MHR24**, inoculated; **plant + MHR24 + M**, inoculated and stained with pyrene marker.

In this regard, further analysis was performed to detect the possible colonization of MHR24 in stem tissue stained with **M**. In order to avoid autofluorescence due to natural pigments that are present in plant tissue, including chlorophylls, lignin, and other compounds present in the cytoplasm and cell wall [27], the stem tissues were treated as in Refs. [28,29]. The marker excess was removed by washing with water, until no fluorescence was detected in the rinsing water under UV lamp irradiation. As can be observed in Figure 11, the control, both uninoculated and inoculated with unstained MHR24, does not present any fluorescence. For the control with **M**, a weak response can be observed in the epidermis. However, the stem tissue inoculated and stained with **M** has a more clearly noticeable fluorescence response in both the epidermis and the vascular bundles, suggesting that MHR24 likely proliferates in the vascular bundles.

## 3. Materials and Methods

The chemical and photophysical properties of pyrene markers **M1–3** in water were previously reported [13]. All the chemicals and reagents were purchased from Sigma-Aldrich, Toluca, State of Mexico, Mexico.

### 3.1. Bacteria Staining Preparation

The *B. amyloliquefaciens* MHR24 rhizobacterium was kindly provided by Dr. Alberto Flores Olivas (Departamento de Parasitología, Universidad Autónoma Agraria Antonio Narro, Saltillo, México). The MHR24 strain was grown routinely in Luria–Bertani (LB) medium on a rotary shaker (150 rpm) at 30 °C up to an O.D. of 0.1 at 600 nm. Later, 1.5 mL of the suspension was centrifuged to obtain a bacterial pellet, which was then resuspended in 1.5 mL of deionized water; the same procedure was performed on samples adjusted to pH 5 and pH 10 with the pH of the deionized water previously modified using 1 M HCl and 1 M NaOH, respectively. A total of 50 µL of the marker (2 mg/mL water) was added and then incubated for 40′ at 30 °C on an orbital shaker (150 rpm). Later, the microtubes were centrifuged to remove residual marker from the supernatant, and the microtubes were resuspended in 1.5 mL of water. Then, 100 µL of the suspension was cast on a microscope slide and left to dry in a sterile laminar flow hood. Control samples were also prepared using the same procedure, but without the pyrene markers.

### 3.2. Characterization

Optical properties of the samples were obtained in acclimated rooms at 17 ± 1 °C. Absorption spectra were recorded on an Agilent Cary 60 UV–Vis (Santa Clara, CA, USA) spectrophotometer by using a clean slide for the baseline. Fluorescence spectroscopy was realized on a Horiba PTI Quantamaster QM-8450-22-c (Burlington, ON, Canada). Spectra were corrected for the detector response, and both excitation and emission slits were fixed at 5 nm for all the samples, which maintains the counts under the linear detection range (<10^6^ counts in the uncorrected spectra). The excitation wavelength for obtaining the emission spectra was 10 nm below the absorption maximum, and the emission maximum was then fixed in the scanning for excitation spectra. Spectra were obtained on the same day, and a clean slide was used as a reference sample, using a proper slide sample holder.

The same samples were also analyzed by LSCM with a Pascal 5 (Carl Zeiss Microscopy, Jena, Germany) microscope in a two-channel (fluorescence and reflection) mode. All the samples were analyzed on the same day and under the same conditions. The excitation was obtained with an Ar laser (488 nm, 200 mW, 50% of power) and a pinhole aperture of 600 μm. Additionally, an AFM (EasyScan 2, Nanosurf AG, Liestal, Switzerland) was used to analyze the topography of the samples. Measurements were realized in air at room temperature (≈20 °C) in contact mode with a PPP-XYCONTR probe (NanoSensors^TM^, Neuchatel, Switzerland). The nominal cantilever force constant was 0.77 N/m. First, a reference map of approximately 80 × 80 μm^2^ was scanned to localize the well-separated bacteria. Then, zoomed scans were performed at 20 × 20 μm^2^ (1024 × 1024 points) at a scan rate of 1 Hz. The surface roughness and profile were obtained from the acquired topography images with the free WSxM Beta 9.3 software [30].

The net bacterial cell surface charge of MHR24 stained with the fluorescent marker **M** was assessed using a zeta potential analyzer (ZETA-check by Microtrac, Montgomeryville, PA, USA). Zeta potential measurements were performed in triplicate at 25 °C. The bacteria without the fluorescent marker and a suspension of just **M** in deionized water were included for comparison.

### 3.3. Molecular Docking Analysis

The outer membrane protein assembly factor BamB of family protein *B. amyloliquefaciens* (GenBank accession no. XZA56257) was obtained from the National Center for Biotechnology Information (NCBI) database in FASTA format. Afterwards, the protein structure was constructed in the Robetta server [31], including consecutive refinement of the receptor. Ligand binding sites were predicted using two independent tools: PrankWeb [32], a machine learning-based server, and I-Tasser [33]. Both methods yielded similar results, which were used to identify the active binding sites for docking.

Molecular docking was carried out with Gnina v1.1 [34]. A grid box of 20 × 20 × 20 Å was centered on the predicted binding site at coordinates (30.0310, −3.9952, −14.5039). The protein was kept rigid, while the ligands were allowed to rotate. A total of ten simulations were run with an exhaustiveness of 128 using the Vinardo scoring method.

The docked complex with the lowest Gibbs energy was chosen to achieve further analysis in Discovery Studio 2025 [35] and PLIP software [36]. Prior to docking, the ligand (in our case, **M**) structure was optimized in Orca version 6.1.0 [37] at the B3LYP/def2-TZVP level of theory.

### 3.4. Plant Growth Experiments at Growth Chamber Conditions

Three experiments were conducted in growth chambers to evaluate the effects of inoculation with *B. amyloliquefaciens* MHR24 on tomato (*Solanum lycopersicum* L.) foliage. The tomato seeds were germinated directly in pots containing sterile peat moss/perlite at a 70/30 (*v*/*v*) ratio for 5 days at 24 ± 2 °C in the dark. Later, 20 days after germination, tomato leaves were inoculated with MHR24 at 1 × 10^8^ UFC/mL. The treatments were performed by mechanical wounding of cotyledons; leaflets were submerged and infiltrated with bacterial suspension, and control plants were treated with sterile water in the same way. The tomato pots were covered with plastic bags for 24 h to increase relative humidity, and the bags were then removed. The tomato plants were incubated at 25 ± 2 °C with a 16 h light and 8 h dark cycle in a growth chamber and fertigated twice with 20N-20P-20K (FertiDrip, AgroDelta, Monterrey, Nuevo León, Mexico).

### 3.5. Stem Staining with ***M*** Pyrene Marker

After 20 days of inoculation, young stems of inoculated plants were treated with a cleaning solution composed of ethanol (95%) and acetic acid (99%) in a 3:1 (*v*/*v*) ratio in order to eliminate autofluorescence interference. The tissue was soaked in cleaning solution until the characteristic green color was completely lost [28,29]. Once bleached, the samples were sliced with a scalpel, placed into a 2.0 mL microtube, and 1.5 mL of sterile water and 50 µL of **M** marker were added. The microtubes were incubated at 30 °C for 15 h at 120 rpm. The samples containing the marker were washed by immersion in water, until the rinsing water did not present excess fluorescence under a UV lamp. Then, microscope slides were prepared for each treatment: **plant control**, **plant + M**, **plant + MHR24**, and **plant + M + MHR24**. Later, the samples were analyzed by LSCM with the Zeiss Pascal microscope on the same day and under the same conditions: excitation with an Ar laser (488 nm, 200 mW, 50% of power) and a pinhole aperture of 1000 μm.

## 4. Conclusions

In this work, the 8-hydroxypyrene-1,3,6-trisulfonic acid trisodium salt (**M**) marker is reported as a suitable fluorescent molecule for staining the PGPR strain of *Bacillus amyloliquefaciens* MHR24, under both in vitro and *in planta* conditions, specifically for its visualization in the phyllosphere through confocal microscopy at 488 nm excitation. The target interactions are multiple, involving hydrogen bonds, electrostatic interactions, and π-stacking, as determined by molecular docking with the simulated *B. amyloliquefaciens* BamB family protein, resulting in a stable protein–marker complex which reflects an increase in the average height and roughness of the bacterial surface as determined by AFM. However, the tomato plant symptoms in the phyllosphere following MHR24 inoculation may indicate a detrimental effect on plant health; therefore, further studies are warranted considering the previously reported biocontrol and biofertilizer properties attributed to the MHR24 strain. Nonetheless, based on the results of this study, its application should be restricted to the rhizosphere.

## Data Availability

The data obtained during the bioassays are available from the corresponding author upon reasonable request.

## Supplementary Materials

The following supporting information can be downloaded at: https://www.mdpi.com/article/10.3390/ijms27094013/s1.

## Abbreviations

The following abbreviations are used in this manuscript:

AFM	Atomic Force Microscope
ESPT	Excited state proton transfer
GFP	Green Fluorescent Protein
ILE	Isoleucine
LB	Luria–Bertani
LEU	Leucine
LSCM	Laser Scanning Confocal Microscopy
M	8-hydroxypyrene-1,3,6-trisulfonic acid trisodium salt (pyrene)
M1	Pyrene replacing the -OH group (8-position) with undecanoic acid chain
M2	Pyrene replacing the -OH group (8-position) with undecane acid chain
M3	Pyrene replacing the -OH group (8-position) with propargyl chain
MHR24	*Bacillus amyloliquefaciens* MHR24
NCBI	National Center for Biotechnology Information
NTR	Nitroreductase
PGPR	Plant growth-promoting rhizobacteria
PY79	*Bacillus subtilis* PY79
*R_q_*	Square roughness
SER	Serine
TRP	Tryptophan
