# Supplementary material for: Fluorescence Detection of Pyrene-Stained Bacillus amyloliquefaciens MHR24 in Tomato (Solanum lycopersicum L.) Stem Tissues
Source: Int J Mol Sci. 2026 Apr 30;27(9):4013. doi: 10.3390/ijms27094013 (PMC13163631; doi:10.3390/ijms27094013)
Supplement: Supplementary file 1 [file ijms-27-04013-s001.zip › ijms-4255237-supplementary.pdf]

Fluorescence detection of pyrene-stained *Bacillus amyloliquefaciens* MHR24 in tomato (*Solanum lycopersicum* L.) stem tissues

Mónica Hernández-Rodríguez[1], Gleb Turlakov[1], Celín Lozano[1], Eduardo Arias[1], Alberto Flores-Olivas[2], Ivana Moggio[1*], and José Humberto Valenzuela-Soto[3*]

Supplementary Materials

[1] Centro de Investigación en Química Aplicada, Departamento de Materiales Avanzados, Boulevard Enrique Reyna Hermosillo 140, Saltillo, 25294, Coahuila, México; monica.hernandez.ps@ciqa.edu.mx (M.H.-R.), gleb.turlakov@ciqa.edu.mx (G.T), celin.lozano.ps@ciqa.edu.mx (C.L.), eduardo.arias@ciqa.edu.mx (E.A.), ivana.moggio@ciqa.edu.mx (I.M).

[2] Universidad Autónoma Agraria Antonio Narro, Departamento de Parasitología, Calz. Antonio Narro, Buenavista, Saltillo, 25315, Coahuila, México; aflooli50@gmail.com (A.F.-O.).

[3] SECIHTI-Centro de Investigación en Química Aplicada, Departamento de Biociencias y Agrotecnología, Boulevard Enrique Reyna Hermosillo 140, Saltillo, 25294, Coahuila, México; humberto.valenzuela@ciqa.edu.mx (J.H.V.-S).

* ivana.moggio@ciqa.edu.mx (I.M); Tel.: 844 438 9830 Ext. 1379; humberto.valenzuela@ciqa.edu.mx (J.H.V.-S); Tel.: 844 438 9830 Ext. 1397.

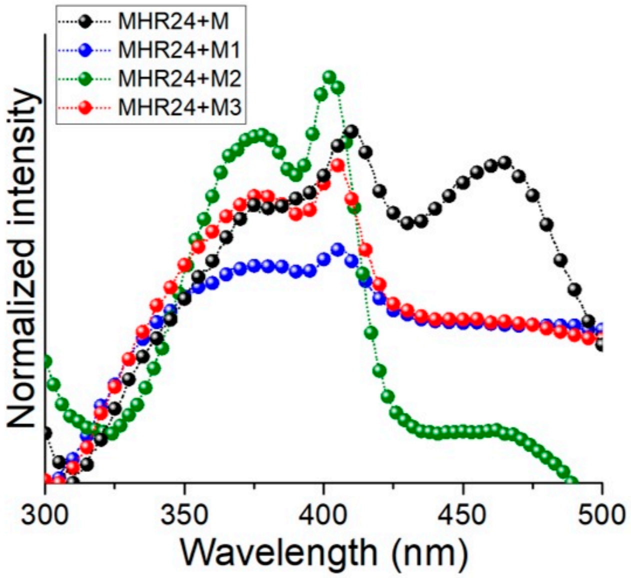

Figure S1. Excitation spectra of pyrene markers **M-M3** in the presence of *Bacillus amyloliquefaciens* MHR24 bacteria when deposited by casting (samples for confocal microscope analysis).

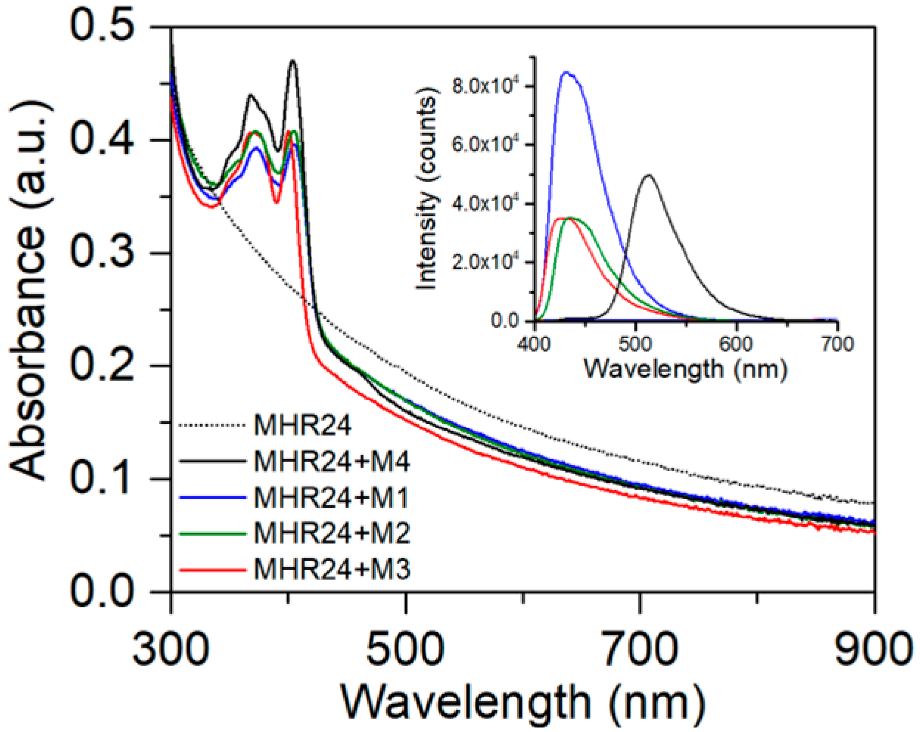

Figure S2. UV-Vis and (inserted) fluorescence spectra of water suspension of pyrene markers **M-M3** in the presence of MHR24 strain. MHR24 spectra are also included for the sake of comparison.

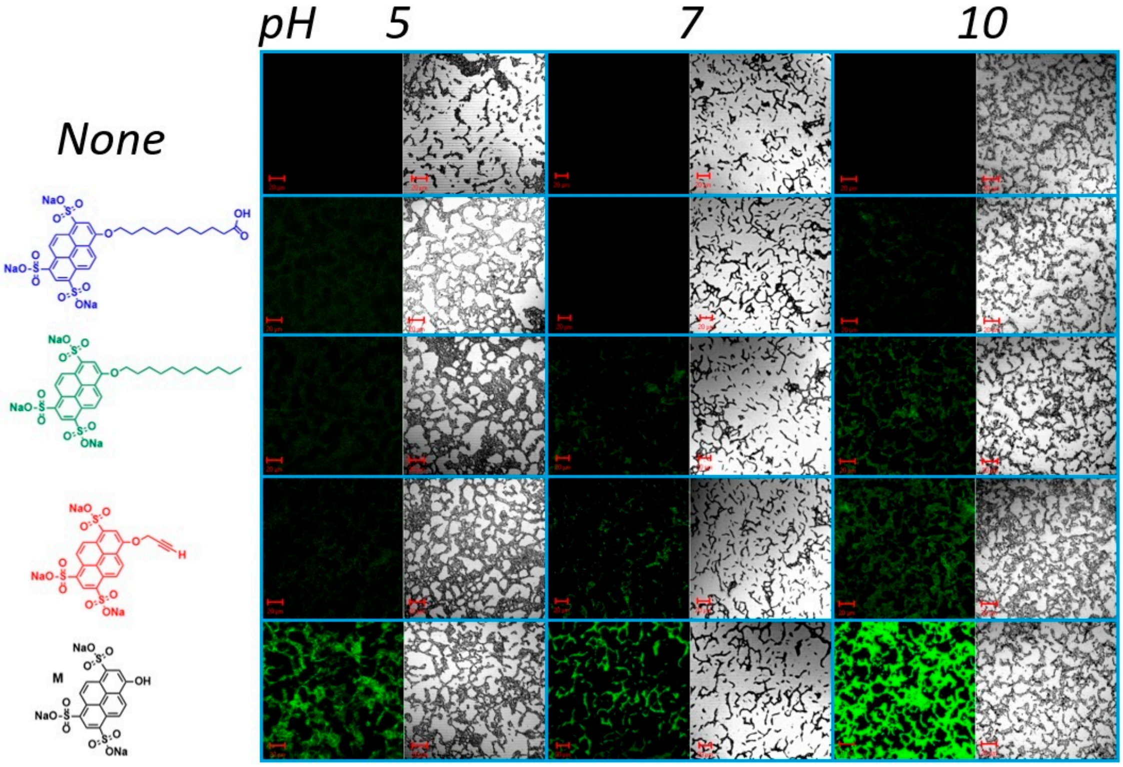

Figure S3. LSCM images of MHR24 strain stained with the four pyrene markers (see inserted chemical structure) at different pH. Fluorescence (left images) and reflection (right images). Excitation of Ar line at 488 nm.

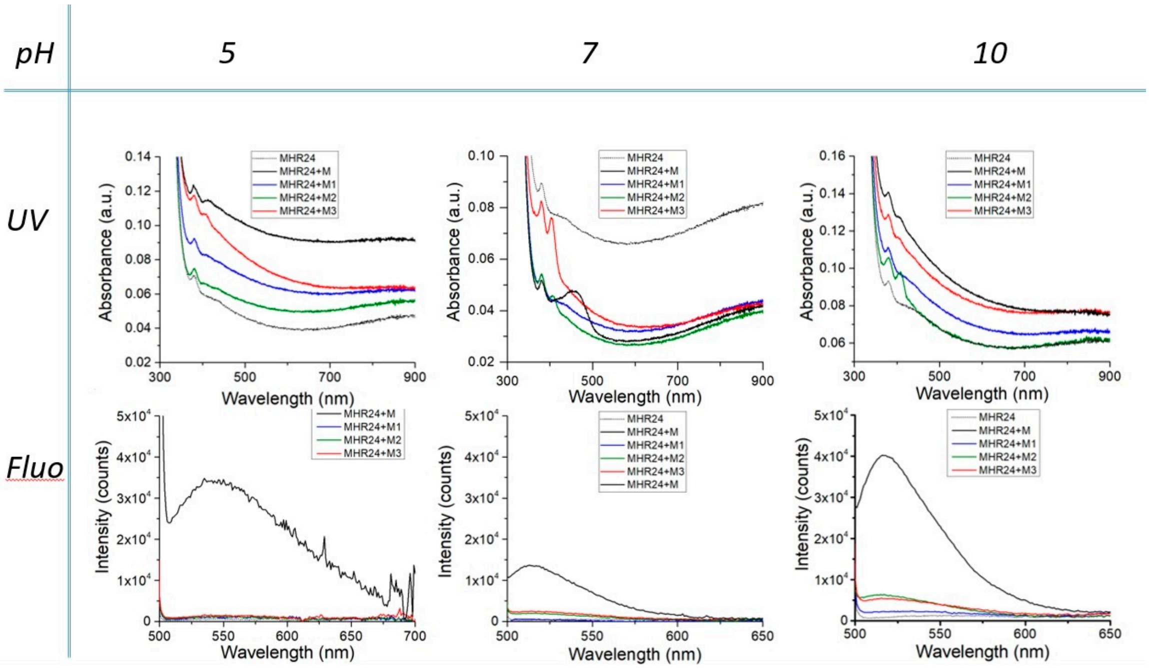

Figure S4. UV-Vis and fluorescence spectra of MHR24 strain stained with the four pyrene markers (see inserted chemical structure) at different pH. Fluorescence (left images) and reflection (right images). Excitation of Ar line at 488 nm.

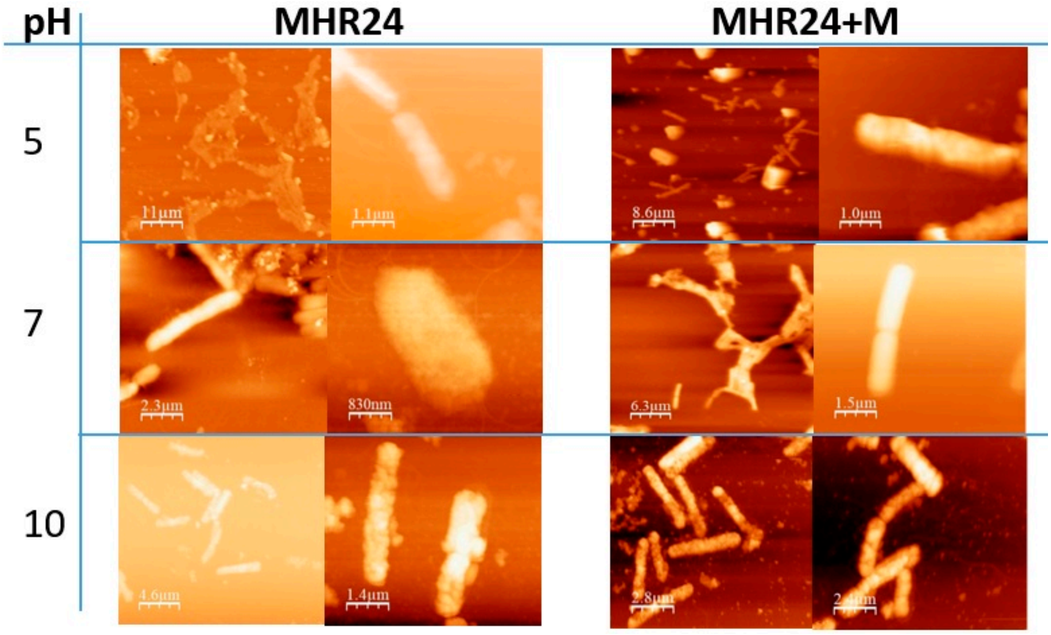

Figure S5. AFM image of MHR24 strain (stained or not) at different pH.

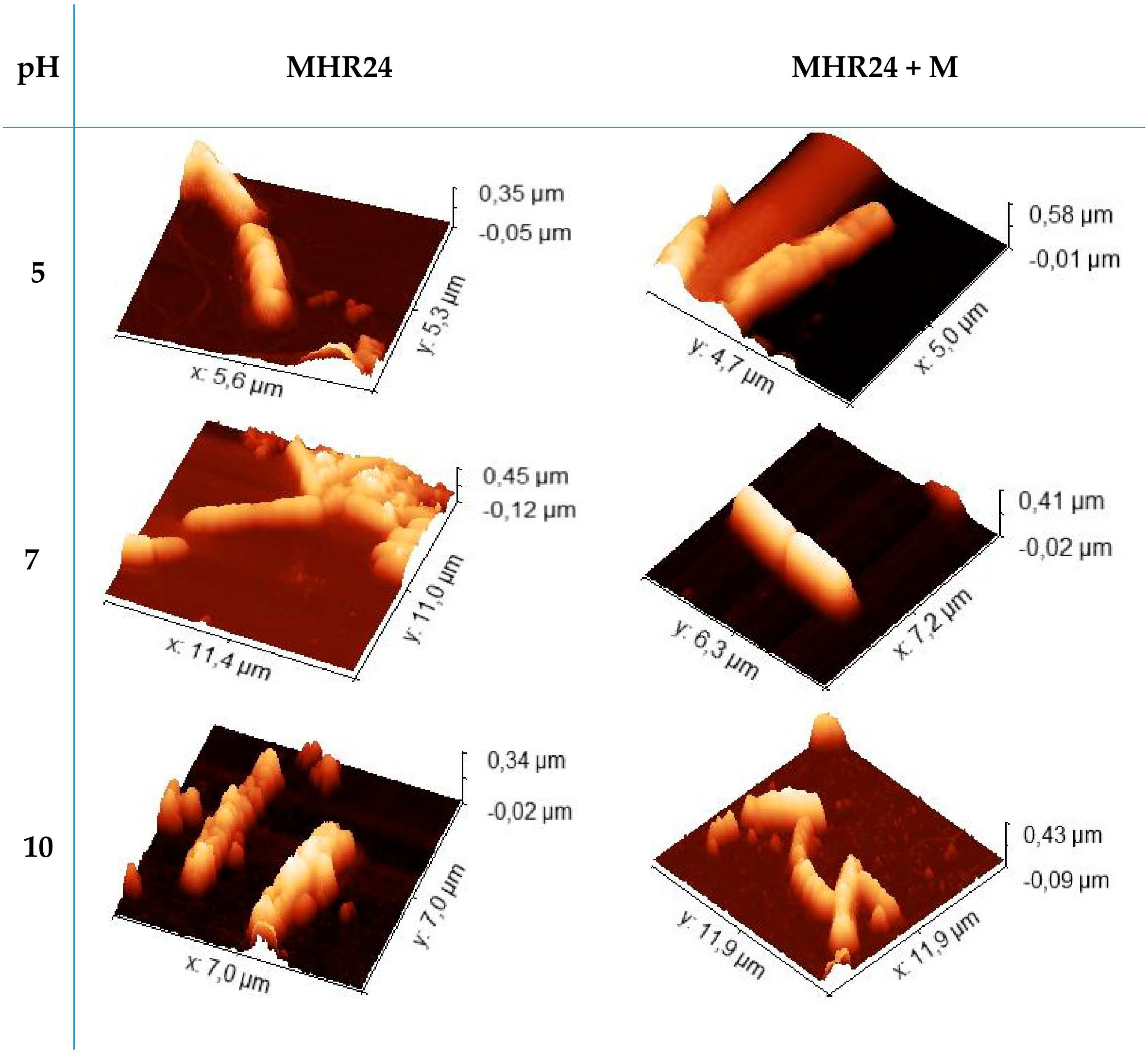

Figure S6. AFM image of profile MHR24 strain stained and not stained at different pH.

pH	MHR24
5	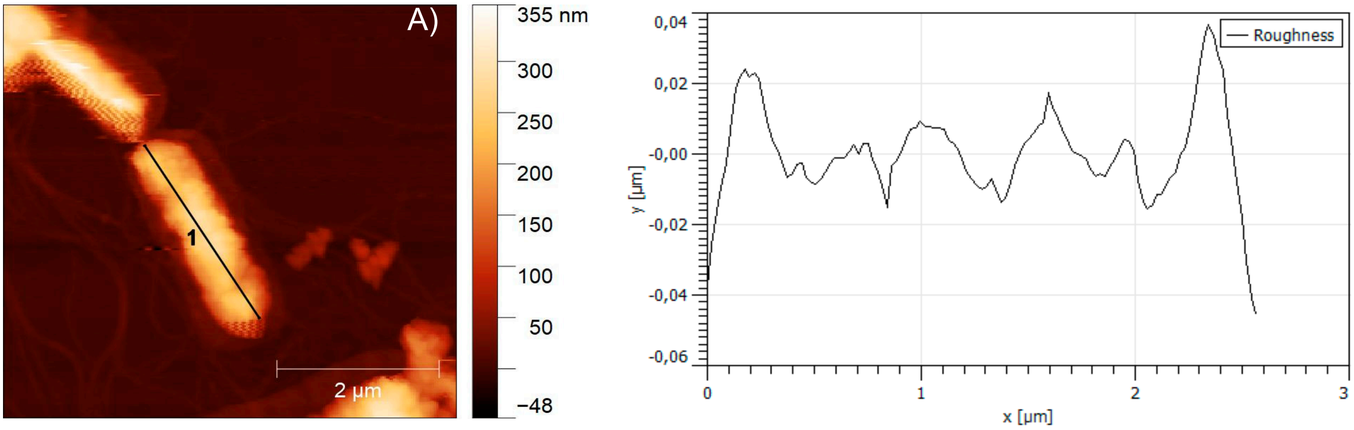
7	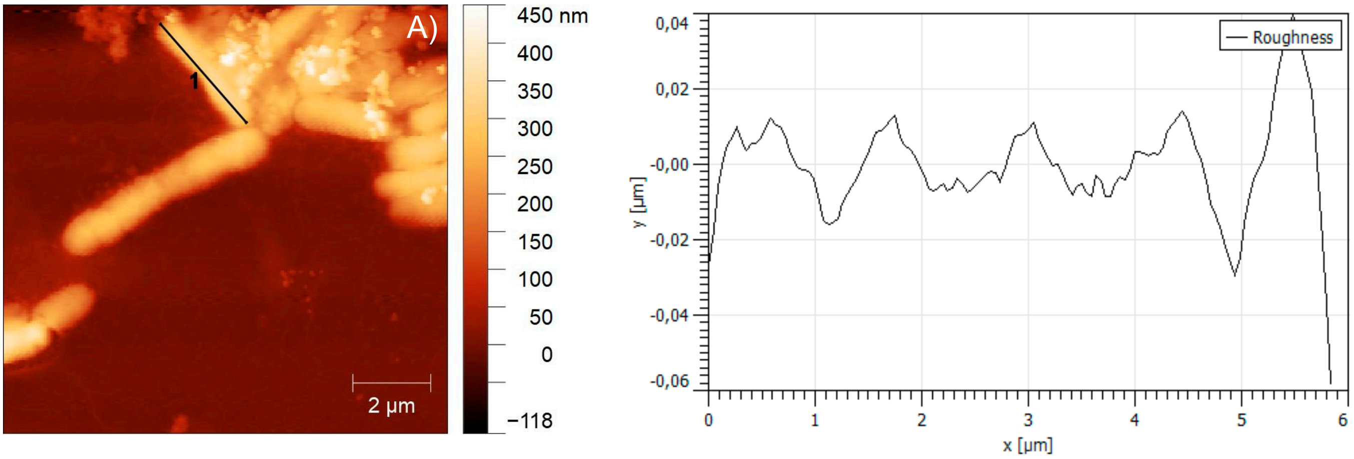
10	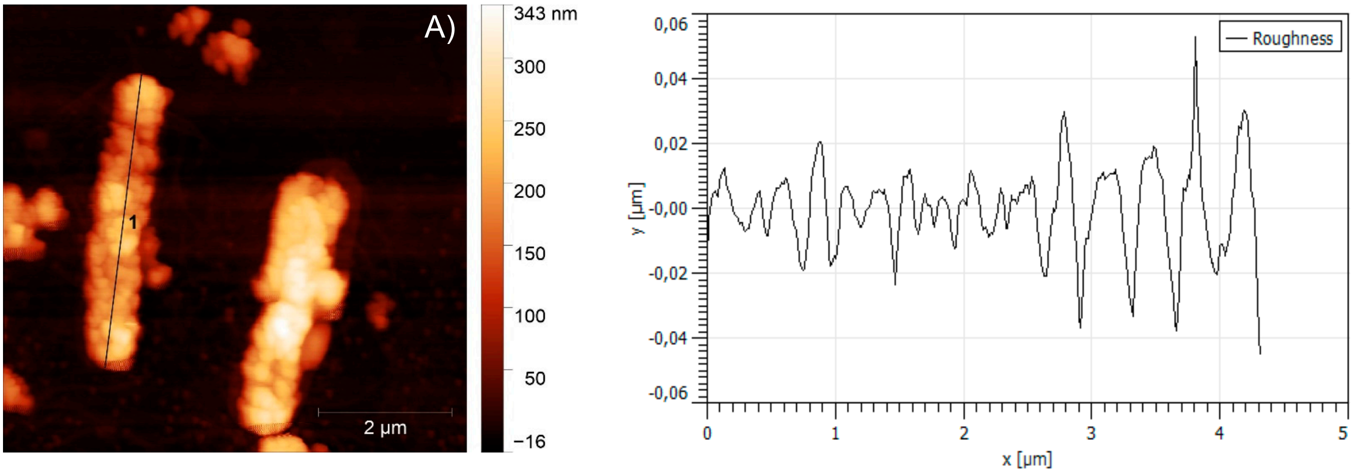

Figure S7. Roughness values obtained from the profile indicated by the black line in A corresponding to each treatment of bacterial pristine at different pH.

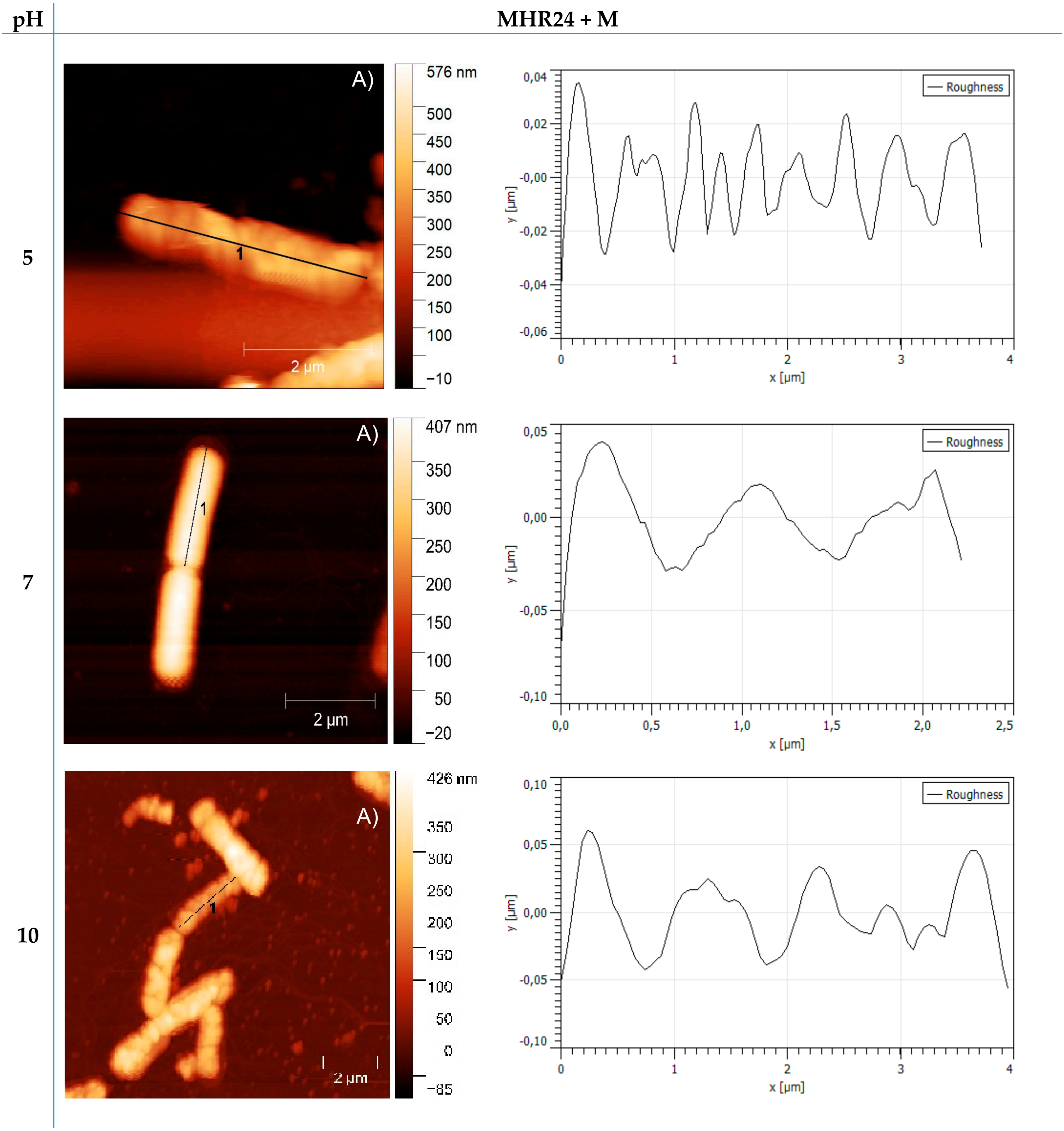

Figure S8. Roughness values obtained from the profile indicated by the black line in A corresponding to each treatment of stained bacteria with M at different pH.

Table S1. Roughness values obtained for MHR24 strain (stained or not) at different pH.

Roughness / pH	MHR24			MHR24 + M		
	5	7	10	5	7	10
Ra	9.39 nm	9.34 nm	9.76 nm	13.85 nm	15.63 nm	24.10 nm
Rq	13.34 nm	13.21 nm	13.01 nm	16.76 nm	19.64 nm	28.98 nm
Rt	81.84 nm	98.08 nm	97.75 nm	110 nm	110.4 nm	135.3 nm
